# A Comprehensive Update on the Chylomicronemia Syndrome

**DOI:** 10.3389/fendo.2020.593931

**Published:** 2020-10-23

**Authors:** Ronald B. Goldberg, Alan Chait

**Affiliations:** ^1^ Departments of Medicine, Biochemistry and Molecular Biology, University of Miami Miller School of Medicine, Miami, FL, United States; ^2^ Department of Medicine, University of Washington, Seattle, WA, United States

**Keywords:** chylomicrons, triglycerides, pancreatitis, lipoprotein lipase, diabetes

## Abstract

The chylomicronemia syndrome is characterized by severe hypertriglyceridemia and fasting chylomicronemia and predisposes affected individuals to acute pancreatitis. When due to very rare monogenic mutations in the genes encoding the enzyme, lipoprotein lipase, or its regulators, APOC2, APOA5, GPIHBP1, and LMF1, it is referred to as the familial chylomicronemia syndrome. Much more frequently, the chylomicronemia syndrome results from a cluster of minor genetic variants causing polygenic hypertriglyceridemia, which is exacerbated by conditions or medications which increase triglyceride levels beyond the saturation point of triglyceride removal systems. This situation is termed the multifactorial chylomicronemia syndrome. These aggravating factors include common conditions such as uncontrolled diabetes, overweight and obesity, alcohol excess, chronic kidney disease and pregnancy and several medications, including diuretics, non-selective beta blockers, estrogenic compounds, corticosteroids, protease inhibitors, immunosuppressives, antipsychotics, antidepressants, retinoids, L-asparaginase, and propofol. A third uncommon cause of the chylomicronemia syndrome is familial forms of partial lipodystrophy. Development of pancreatitis is the most feared complication of the chylomicronemia syndrome, but the risk of cardiovascular disease as well as non-alcoholic steatohepatitis is also increased. Treatment consists of dietary fat restriction and weight reduction combined with the use of triglyceride lowering medications such as fibrates, omega 3 fatty acids and niacin. Effective management of aggravating factors such as improving diabetes control, discontinuing alcohol and replacing or reducing the dose of medications that raise triglyceride levels is essential. Importantly, many if not most cases of the chylomicronemia syndrome can be prevented by effective identification of polygenic hypertriglyceridemia in people with conditions that increase its likelihood or before starting medications that may increase triglyceride levels. Several new pharmacotherapeutic agents are being tested that are likely to considerably improve treatment of hypertriglyceridemia in people at risk.

## Introduction

The chylomicronemia syndrome (CS) is a term that is used to describe individuals with either intermittent or persistent fasting chylomicronemia causing severe hypertriglyceridemia (HTG). It is usually defined as being present when triglyceride values exceed 1,000 mg/dl (1) or 10 mmol/L (885 mg/dl) (2), when the risk for pancreatitis is increased. Fasting chylomicronemia may rarely be due to a monogenic disorder that markedly reduces the activity of lipoprotein lipase (LPL), resulting in decreased clearance of the triglyceride-rich lipoproteins (TRL) from plasma. This is referred to as the familial chylomicronemia syndrome (FCS) and is very resistant to treatment. However most cases with CS occur in individuals with a polygenic form of hypertriglyceridemia (HTG) that typically manifests with moderately elevated triglyceride levels (500–999 mg/dl), in whom severe HTG and CS may develop as a result of the underlying genetic tendency being complicated by one or more conditions or medications that aggravate HTG and is termed the multifactorial chylomicronemia syndrome (MFCS) (3). In the NHANES cohort of 5,680 adults representative of the US population, the prevalence of moderate to severe hypertriglyceridemia (500–2,000 mg/dl) was 1.7% (4) and the CS has been estimated to occur in 1:600 of the population (4). MFCS will typically respond to treatment or removal of the secondary cause or causes together with triglyceride lowering medications. Because the principal health risk of the CS is pancreatitis, which can be prevented if triglyceride levels are maintained below 500 mg/dl, clinical recognition of polygenic HTG and the factors that can aggravate it are key to the prevention of much of the morbidity and mortality associated with the CS and is the major focus of this review.

### Triglyceride Dysmetabolism and Development of the Chylomicronemia Syndrome

Triglycerides are synthesized and secreted by the liver in very low density lipoprotein (VLDL) as well as from the intestine after fat digestion and absorption in chylomicrons. These TRL are cleared from the circulation by lipoprotein lipase (LPL) located in adipose tissue and muscle capillaries where the hydrolyzed fatty acids are either stored or used as fuel. Normally chylomicrons are cleared from the circulation by 3 to 4 h after a meal while VLDL are continuously secreted by the liver. The clearance of TRL becomes saturated at triglyceride levels of about 500 to 700 mg/dl such that additional TRL entering the circulation cannot be readily be removed by LPL (5). Because a single meal may deliver over 30 to 50 g of triglyceride into the circulation in chylomicrons, under conditions when chylomicrons accumulate excessively, they may continue to be present even after an overnight fast causing fasting chylomicronemia and the CS. Typically, in these circumstance triglyceride values exceed 1,000 mg/dl and eruptive xanthomas and lipemia retinalis may develop. After hydrolysis of VLDL and chylomicrons by LPL, the remnant particles which still contain significant amounts of triglyceride, are cleared or metabolized by the liver.

Overproduction as well as reduced clearance of TRL may contribute to the CS (3). LPL is synthesized mainly in adipocytes and myocytes as an immature protein and as it matures under the influence of lipase maturation factor 1 (LMF1) and secreted into the interstitial space, it is transported transendothelially and tethered to an anchoring protein, glycosylphosphatidyinositol high density lipoprotein binding protein 1 (GPIHBP1) on the luminal surface of the endothelium, where it is available for binding to circulating TRL and hydrolysis of its triglyceride molecules (6, 7). LPL activity is regulated by several TRL apolipoproteins. These include APOC2, an essential cofactor for LPL activity, APOA5, which is thought to stabilize the LPL-TRL complex, and APOC3, which inhibits LPL activity. In the process of TRL hydrolysis, APOE and APOC3 bound to the remnant particles, respectively activate or inhibit their hepatic clearance (7). Recent findings reveal that a group of angiopoietin-like proteins which includes ANGPTL3 produced by the liver inhibits LPL in peripheral tissues in an endocrine fashion, and ANGPTL4, which is produced in several tissues, inhibits LPL in a paracrine fashion, retarding clearance of the TRL. ANGPTL8 also plays a role in inhibiting LPL and seems to facilitate the ability of ANGPTL3 to inhibit LPL. These ANGPTL proteins are regulated by both local and systemic nutritional and endocrine factors, providing a link between energy metabolism and LPL activity, TRL clearance and free fatty acid uptake. LPL activity regulators, which are increasingly being demonstrated to play a role in the genesis of HTG, constitute targets for novel treatment strategies for HTG.

## Causes of the Chylomicronemia Syndrome

### Monogenic Disorders of Lipoprotein Lipase Activity—Familial Chylomicronemia Syndrome (FCS)

This rare group of autosomal recessive disorders are usually due to homozygous or compound heterozygous mutations of the *LPL* gene leading to non-function or very low function of LPL and have an estimated population frequency of 1 per million (7). Even rarer causes are loss-of-function mutations in *APOC2, APOA5, GPIHBP1 and LMF1* genes (7). Triglyceride levels tend to be higher in those with *LPL* mutations than in those with *non-LPL* FCS. Patients with FCS are younger and less likely to have any of the aggravating factors for HTG compared to those with MFCS, but are more likely to develop pancreatitis probably because of life-long, sustained chylomicronemia (8). They are less like to have cardiovascular disease (CVD) than those with MFCS because of the severe reduction in LPL activity, which reduces atherogenic chylomicron and VLDL remnant formation and accumulation which may occur in MFCS (7). This feature formed the basis for the original terminology used to describe these patients, namely Frederickson Type 1 hyperlipoproteinemia. The implication of this difference is that accumulation of atherogenic remnants in MFCS but not FCS has consequences for heightened CVD risk in those with MFCS.

### Polygenic Causes of Hypertriglyceridemia—Multifactorial Chylomicronemia Syndrome (MFCS)

This group of disorders are the most frequent cause of the CS, occurring in about 1;600 of the population (3, 7, 9, 10). They are thought to result from the clustering of multiple genetic variants that include both rare heterozygous variants of the *LPL, APOC2, APOA5, APOB, GCKR, LMF1, and GPIHBP1* genes and more frequent variants with small effects in ~40 genes. Recently*, CREB3L3*, the gene for the transcription factor cyclic AMP–responsive element–binding protein H (CREBH), which is induced by metabolic cues such as fasting and fatty acids and leads to increases in APOC2 and APOA5 and reduction in APOC3 production has been shown to be associated with MFCS (10). In most cases these genetic variants are thought to require an aggravating effect of one or more secondary conditions or medications that exacerbate the metabolic defect leading to saturation of clearance mechanisms to cause the CS. In a recent survey of the genetic determinants of severe HTG in 567 subjects, 1.1% had homozygous or compound heterozygous rare variants (FCS), 14.4% had heterozygous rare variants among which heterozygous mutations of the *LPL, APOA5 and CREB3L3* genes were most frequent, 32.0% had an extreme accumulation of common variants and 52.6% remained genetically undefined (10). Common genetic variants influence triglyceride levels in the general population and the presence of multiple common variants, each with small effects in an individual, and constituting a polygenic risk score, interacts with secondary factors to cause mild to moderate HTG, the clinical phenotype of polygenic HTG (11). The phenotype tends to cluster in families giving rise to a familial pattern of HTG although there is no established cut-off in triglyceride level to distinguish these individuals from the rest of the population except those established by convention to delineate HTG (>150 mg/dl). The entities of familial hypertriglyceridemia and familial combined hyperlipidemia, once thought to be monogenic disorders and both known to be associated with the development of the CS, are more likely due to polygenic disorders involving multiple genetic variants affecting APOB-lipoprotein metabolism (11). Mild to moderate HTG also occurs in subjects with familial dyslipoproteinemia due to mutations in the *APOE* gene (6) where retarded clearance of remnant lipoproteins can cause HTG. Since the risk for developing severe HTG and the CS is due to the concurrence of one or more secondary factors that aggravate the underlying HTG and since most of these develop or occur later in life, the MFCS typically presents in mid- to late adulthood although it may occasionally develop in childhood.

### Familial Partial Lipodystrophy

Congenital/familial and acquired lipodystrophies are rare conditions that predispose to the development of the CS because of the prevalence of severe HTG in these individuals (12). Although the pathophysiology of HTG has not been well studied in these subjects, the reduced ability to deposit free fatty acids in adipose tissue due to its maldevelopment, and accelerated lipolysis are thought to play mechanistic roles (13). The two most common forms of familial partial lipodystrophy are the Dunnigan (type 2) and Köbberling (type 1) varieties. Mutations in the *LMNA* and less frequently the *PPARG* genes are seen in individuals with the Dunnigan phenotype (13). No mutations in single genes have so far been described for the Köbberling form, the phenotype which has many features in common with the metabolic syndrome (14, 15), and which is grossly underdiagnosed. However, there are several diagnostic features that distinguish it from the metabolic syndrome (12, 14, 15). Both forms can present with severe insulin resistant diabetes, severe HTG and non-alcoholic fatty liver disease.

### Other

#### Autoimmune Hyperlipidemia

Severe HTG may rarely develop as a result of autoimmune hyperlipidemia without the need for the presence of other aggravating factors. The original descriptions were in patients with myeloma sometimes associated with tuberous or planar xanthomas and in most cases the antibodies were found to react with VLDL and other lipoproteins or with heparin (16). Subsequently reports of autoimmune hyperlipidemia have been described in patients with lupus erythematosus and Sjögren’s syndrome and in several cases antibodies to LPL were identified. More recently 6 cases with CS were found to have antibodies to GPIHBP1, four of whom had either lupus or Sjögren’s syndrome (17) attesting to the importance of this protein for LPL activity. In addition among 33 patients with unexplained CS and no genetic mutations, one was found to have GPIHBP1 autoantibodies (18) and in another recent report a patient with newly acquired CS resistant to standard treatment was found to have GPIHBP1 antibodies that disappeared with normalization of HTG after treatment with the anti-B cell monoclonal antibody rituximab (19). These findings point to the “GPIHBP1 autoantibody syndrome” (17) as a novel form of autoimmune hyperlipidemia. Moreover, GPIHBP1 autoantibodies were identified as the cause of the CS in a patient treated with interferon for multiple sclerosis (20). Interferon is known to increase the risk of autoimmune disease and discontinuation of the interferon caused the antibody to disappear. Interferon does increase triglyceride levels modestly, attributable to increased hepatic lipogenesis and decreased LPL activity (21), but it is unknown whether the small percentage of individuals receiving interferon who develop severe HTG (22) do so because of the occurrence of GPIHBP1 antibodies.

#### Glycogen Storage Disease

Moderate to severe HTG is common in glycogen storage disease type 1 (glucose-6-phosphatase deficiency) and is attributed to shunting of the increased glucose-6-phosphate into hepatic lipogenesis and increased VLDL levels. CS is uncommon. Post-heparin LPL activity does not appear to be decreased (23). In a series of 230 cases with classical glycogen storage disease type 1, 72.7% had triglyceride levels >3 mmol/L (>270 mg/dl) but only 3 individuals had a history of pancreatitis (24).

## Clinical Conditions and Medications Associated With Development of the Chylomicronemia Syndrome

Although many conditions and drugs may aggravate polygenic HTG, surveys of the acquired factors present in cohorts with severe HTG or with HTG pancreatitis have highlighted the importance of overweight and obesity, diabetes, excessive alcohol consumption, and chronic renal disease, often complicated by triglyceride-raising medications (25–37). Demographic and sociocultural factors have important effects as well (36). It is important to identify any and all modifiable aggravating factors because dietary and currently available pharmacologic interventions to lower triglyceride levels in individuals with CS may not be sufficient unless aggravating factors are removed or minimized, particularly over time. Other than the rare cases of FCS, FPLD and autoimmune hyperlipidemia, which can have persisting chylomicronemia without the need for additional aggravating factors, nearly all patients presenting with the CS have secondary aggravating factors (9). Thus, effective management of the CS is should encompass a prevention strategy to identify the secondary conditions or medications that may aggravate pre-existing HTG. Given the importance of aggravating factors to the development of the CS, these are discussed in detail below (**Table 1**).

**Table 1:** Clinical conditions and medications associated with development of the chylomicronemia syndrome.

Clinical conditions	Diabetes
	Obesity
	Alcohol excess
	Chronic kidney disease
	Nephrotic syndrome
	Pregnancy
	Hypothyroidism
Medications	Diuretics
	Antihypertensives
	Estrogen and estrogen receptor agonists
	Corticosteroids
	Protease inhibitors
	Antipsychotics and antidepressants
	Immunosuppressives
	Retinoids
	Propofol
	L-asparaginase

### Clinical Conditions Most Commonly Associated With the Development of the Chylomicronemia Syndrome

#### Diabetes

Based on a representative US sample, 22.5% of people with diabetes have triglyceride levels >200 mg/dl and those with HbA1c >7.0% have higher triglyceride levels than those with HbA1c < 7.0% (38), although relatively few have triglyceride levels >500 mg/dl and even fewer >1,000 mg/dl. The HTG is thought to be due to TGL overproduction in type 2 diabetes as a result of increased release of free fatty acids from adipose tissue, enhanced hepatic lipogenesis and increased secretion of large TRL particles by the liver as well as the intestine as a consequence of insulin resistance and hyperglycemia (39). Although LPL activity is known to be decreased in insulin deficient states (40), recent developments in the understanding of LPL regulation have also shown that insulin resistance leads to increased APOC3 and ANGPTL4, which reduce LPL activity and TGR remnant particle clearance (41–43).

The prevalence of diabetes in reports of cohorts with severe HTG varies widely, ranging from 25% to 76% in reported studies (25–37). In a recent large US County Health System survey of risk factors for very severe HTG (triglyceride levels >2,000 mg/dl) in whom most of the identified patients were Hispanic, diabetes was present in 76% (78 out of 103 cases) and was also frequently present in those with other secondary factors, such as obesity, excessive alcohol intake and use of triglyceride-raising drugs (25). The patients with diabetes were said to be uncontrolled with a median HbA1c of 10.4% [interquartile range 8.2–12.6%], 3 out 78 had type 1 diabetes and 21 had diabetic ketoacidosis. Although the majority of patients with diabetes and severe HTG have type 2 diabetes, severe HTG may occur in poorly controlled insulin deficient diabetes (44), where the combination of LPL deficiency and continued dietary fat intake are key factors. In a survey of risk factors for severe HTG in which 75% had diabetes, 82% of these were receiving insulin treatment (37).

In an early report, the observation that index patients with triglycerides >2000 mg/dl had considerably higher triglyceride levels than their first- degree relatives with HTG, prompted a search for other potential causes of HTG in the index patients (45). Ninety-four percent were found to have a potential secondary cause, 63% of which was uncontrolled or untreated diabetes. Moreover, in the original report of the CS in diabetes, treatment of the diabetes led to a marked reduction in triglyceride levels, but in no cases did it fall into the normal range (46). This provides support for the notion that the pathophysiological disturbances in triglyceride metabolism that accompany diabetes can interact with polygenic forms of HTG to result in triglyceride levels that exceed the saturation point for triglyceride removal from plasma (47), thereby leading to severe HTG and the MFCS (9).

Overall, the findings support the contention that patients with diabetes who develop HTG-induced acute pancreatitis are typically in poor glycemic control despite being on insulin treatment. However, the extent to which poor glycemic control is a defining feature of concurrent diabetes and severe HTG has not been studied, nor is it known whether the genetic variants presumed to be present in these cases are any different from those in the general population with severe HTG. More detailed studies of the genetic, pathophysiologic and clinical characteristics of these individuals are needed in order to be able to better target at risk subgroups for more effective intervention. Complicating this area is evidence that genetic variants linked to triglyceride levels may be associated with lower glucose levels in the population (48). On the other hand, in a Japanese study the majority of those with severe HTG who developed pancreatitis did not have diabetes (32). It is likely that other risk factors for severe HTG as well as race/ethnicity and sociocultural differences may interact with less severe diabetes in many circumstances. Finally, the extent to which the CS contributes to the increased frequency of acute pancreatitis in diabetes is unclear (49).

#### Obesity

Surveys of severe HTG indicate that up to 50% of patients are obese (25–37, 50). However many of these subjects have other risk factors for HTG such as diabetes or alcohol excess, and obesity is associated with an increased occurrence of biliary disease which is a common cause of pancreatitis, so that the exact role of obesity in the genesis of CS and risk of pancreatitis is unclear. The effect of obesity on triglyceride metabolism is thought to be more strongly related to visceral obesity than to total adiposity (50). Increased release of free fatty acids from visceral fat increase VLDL production especially in the presence of insulin resistance which is present in the majority of obese individuals (51). While LPL is increased in obesity its functional regulation is impaired, with recent evidence suggesting that increased APOC3 and ANGPTL3 in obese individuals may contribute to reduced LPL activity and reduced remnant particle clearance (52). These abnormalities at the least provide the substrate for aggravation of mild to moderate polygenic forms of HTG to exceed the saturation point for triglyceride removal and hence lead to MFCS.

#### Alcohol Excess

Population studies demonstrate that alcohol consumption is linearly associated with plasma triglyceride levels and was 0.37 to 0.46 mmol/l (33–41 mg/dl) higher in subjects drinking more than 5 alcoholic drinks per day (53). In addition, alcohol consumption is more likely to cause HTG in overweight than in normal weight individuals (54). Alcohol has been shown to stimulate VLDL secretion possibly through enhanced assembly of VLDL, combined with elevated hepatic delivery of free fatty acids as a results of enhanced adipose tissue lipolysis (55, 56). In addition, there is evidence for a prolonged postprandial lipemic response due to decreased LPL activity although LPL activity has also been reported to be increased by chronic alcohol intake (57)

Excessive alcohol intake was found to be present in 9% to 50% of individuals with severe HTG and may coexist with obesity and diabetes (25–37) and in studies of the prevalence of secondary factors in cohorts with very severe HTG, excessive alcohol intake was the second most common secondary factor after diabetes (25, 45), accounting for 11% of potential causes of HTG in addition to a familial disorder (45). In a Dutch retrospective survey of subjects with severe HTG, excessive alcohol intake was more likely to be present in those with triglyceride levels >15.9 mmol/L (>1450 mg/dl) and especially >26.8 mmol/L (>2365 mg/dl) (58). Moreover, in patients with alcoholic HTG, triglyceride levels fell to within normal limits after alcohol withdrawal in only 1/5 subjects with triglyceride levels >1000 mg/dl at baseline (59), suggesting that they had an underlying cause of HTG in addition to excess alcohol intake. There is evidence to indicate that the effect of alcohol on triglycerides is influenced by genetic polymorphisms (60–62). These findings are again consistent with the notion that MFCS is a result of the co-existence of an underlying polygenic form of HTG and another potential cause of HTG, in this case, alcohol.

#### Chronic Kidney Disease and Nephrotic Syndrome

Chronic kidney disease is known to be associated with HTG in relation to the rise in serum creatinine and to albuminuria (63). The mechanism for HTG is still somewhat unclear but appears to be due mainly to reduced clearance of TRL and related to elevated APOC3 and the presence of insulin resistance known to accompany chronic renal insufficiency (64). There also is experimental evidence for reduced GPIHBP1 playing a role (65). The prevalence of severe HTG in patients with chronic kidney disease is unknown, although in many reports 5% to 10% of those with severe HTG or HTG-associated acute pancreatitis had chronic renal failure (25–37), possibly as a result of their also having background genetic HTG.

Severe HTG is known to occur in both children and adults with nephrotic syndrome and is thought to be due to reduced LPL activity due to decreased GPIHBP1 and increased ANGPTL4 and APOC3 levels (66). Since high-dose corticosteroids are often used in these patients, this may be a confounding factor, A case report of severe HTG in a child with the nephrotic syndrome was reported to be associated with an APOE mutation (67).

#### Hypothyroidism

Although the typical lipid disorder in hypothyroidism is hypercholesterolemia, elevated triglyceride levels may also occur, and this is thought to result from an increase in ANGPTL3 and ANGPTL8 levels (68). In addition, hypothyroidism is associated with reduced hepatic lipase activity and slowed remnant clearance (69). Triglyceride levels are usually considerably lower than those seen in MFCS, but again the co-existence of hypothyroidism with a polygenic form of HTG could lead to saturation of triglyceride removal mechanisms, leading to severe HTG (9, 25).

### Commonly Used Medications That May Contribute to the Chylomicronemia Syndrome

It is difficult to know how large a role medication that increase triglyceride levels contribute to the development of the CS. Most of the agents described below and in **Table 1** have been implicated in case reports to contribute to the presentation of severe HTG and CS, but surveys or clinical trials of the more widely used drugs usually demonstrate only mild triglyceride raising effects. Based on surveys of risk factors associated with severe HTG and/or hypertriglyceridemic pancreatitis medications are reported to collectively contribute in up 10% of cases in some series (25–37), again suggesting that very high triglyceride levels associated with the use of these drugs results from their use in subjects with a baseline genetic HTG, leading to saturation of triglyceride removal mechanisms, thereby leading to severe HTG. Use of medications that can elevate plasma triglyceride levels accounted for 7% of associated conditions in patients with very severe HTG in the US County Health System report (25). Because many of these medications are relatively widely used drugs, especially in selected patient groups, and given the prevalence of mild to moderate HTG in the population, it is mandatory that clinicians using these agents identify at risk patients with HTG when starting treatment with these agents, particularly if there are other aggravating conditions such diabetes, obesity and alcohol excess or when combinations of these drugs are being used.

#### Diuretics and Antihypertensives

Thiazide diuretics and beta blockers have both been shown to increase triglyceride levels by 0% to 50% in older studies (70, 71) although there is evidence that these effects wane with time (72). The mechanism for the thiazide effect on triglyceride levels is unknown. In the case of beta blockers there is evidence that non-selective beta blockers reduce LPL activity (73, 74). The use of indapamide instead of thiazides and of selective beta blockers with intrinsic sympathetic activity rather than non-selective beta blockers in potentially susceptible individuals are favored alternatives since they have little triglyceride raising potential (75). The relevance of these findings to the development of severe HTG and the CS in patients with HTG who use these agents is unclear particular since both agents have been reported to cause pancreatitis (73, 75). Whether pancreatitis associated with their use is due to severe HTG in these cases is unknown.

#### Estrogen, Estrogen Receptor Agonists, and Pregnancy

Oral estrogen administration increases triglyceride levels 10% to 50% (76). Importantly the effect varies by dose and type of estrogen preparation, is generally smaller with estrogen/progestin combinations (77) and not observed with transdermal estrogen. The increase in triglyceride level is thought to be due to VLDL overproduction resulting from activation of the estrogen receptor (78). It is unclear whether estrogen alters LPL in humans. Although estrogen administration or gestational HTG does not comprise a significant number of cases in surveys of factors causing CS or HTG pancreatitis, there are many case reports of estrogen-induced CS and of HTG pancreatitis and these presumably are due to exacerbation of underlying monogenic or polygenic HTG (79). Given the widespread use of estrogenic compounds in women and in transsexual men and the prevalence of mild to moderate HTG in the population at risk, individuals with baseline HTG should be identified when using these agents. Among estrogen receptor modulators, tamoxifen used in the management of breast cancer as well as clomiphene, used for infertility have both been reported to cause severe HTG and pancreatitis (80, 81), while raloxifene has minimal effects in normotriglyceridemic women (82), but may cause severe HTG in women with a history of estrogen-induced HTG (83). Importantly estrogen therapy is often not discontinued by clinicians in women with a history of HTG-induced pancreatitis (84). Endogenous hyperestrogenemia may cause severe gestational HTG and pancreatitis in women with HTG in the second or third trimesters of pregnancy, where it can be difficult to control, particularly in subjects with monogenic CS or with other aggravating factors such as diabetes (85, 86).

#### Corticosteroids

Although not widely appreciated, corticosteroids raise triglyceride levels modestly (87). Most studies reporting this finding have been in cohorts with chronic steroid-requiring conditions, such as asthma, rheumatoid arthritis and post-transplantation, where findings are often confounded by other factors. The most definitive study was in patients with Cushing’s syndrome who had mild hypertriglyceridemia compared to controls that resolved after curative surgery (88). Kinetic studies revealed that the elevated triglyceride level was due to VLDL overproduction likely due to insulin resistance rather than a removal defect since post-heparin LPL levels were normal. However reports of HTG-induced acute pancreatitis occurring in 2 patients with a history of fibrate-treated HTG receiving short courses of high-dose corticosteroids point to the likelihood that patients with polygenic HTG may be at risk for exacerbation of their HTG following this commonly used treatment in clinical practice (89). This is supported by data from a survey of severe HTG in Japanese subjects in whom 27% of cases were associated with corticosteroid use (32).

#### Protease Inhibitors

Treatment of human immunodeficiency virus with protease inhibitors has been shown to increase triglyceride levels by 22% in a 12 month study, although this does not apply to atazanavir (90). There is evidence for both VLDL overproduction as well as reduced LPL activity (91, 92). In addition, studies have demonstrated an interaction between the triglyceride raising effect and *APOC3* gene polymorphisms (93). There have been case reports of chylomicronemia (94) and of HTG-induced pancreatitis attributable to protease inhibitors (95), but in surveys of drugs associated with severe HTG or HTG-induced pancreatitis, the use of protease inhibitors is not mentioned suggesting that these agents are uncommon causes of CS.

#### Antipsychotics and Antidepressants

Both first and second generation antipsychotics may cause HTG (96). Among atypical antipsychotics, olanzapine and quetiapine increase triglycerides modestly in relation to weight gain and hyperglycemia, with risperidone, ziprasidone, and aripiprazole having milder effects (97). There is evidence that these agents increase hepatic synthesis and triglycerides as well as inhibit LPL (98). Olanzapine and quetiapine have both been reported to cause severe HTG and occasionally pancreatitis (99, 100), presumably when used in subjects with genetic forms of HTG.

Hypertriglyceridemia has also been described as occasionally occurring with antidepressant medications. Perhaps best described is the HTG in patients treated with the tetracyclic antidepressant mirtazapine, in which 6% of patients were reported to have triglyceride levels >500 mg/dl (101) and there are several reports of HTG-associated acute pancreatitis occurring in patients treated with mirtazapine (102).

#### Immunosuppressive Agents

Cyclosporine, a calcineurin inhibitor, and sirolimus and everolimus (also used as a chemotherapeutic and in drug-eluting coronary artery stents), both inhibitors of the mammalian target of rapamycin (mTOR), have all been shown to cause HTG. Tacrolimus has minimal effects on triglyceride levels. The effect of cyclosporine on triglyceride levels is relatively mild, increasing by 50 mg/dl in one study (103), but triglyceride levels were >400 mg/dl in 14% to 25% of patients with sirolimus in one report of renal transplant recipients (104) and in another in 28% of sirolimus and 35% of everolimus treated patients (105). Both sirolimus and everolimus have been associated with the development of severe HTG (106, 107). These agents increase APOC3 levels and inhibit LPL (108, 109).

#### Retinoids

Isotretinoin used in treatment for acne was found to cause mild to moderate and occasionally severe HTG and rarely HTG pancreatitis. As a result its use is largely limited to treatment of severe acne vulgaris. In one study isotretinoin increased mean triglyceride levels by 50% with 17% of individuals having triglyceride values >200 mg/dl. (110). Retinoids increase APOC3 levels but do not appear to directly inhibit LPL in humans (111). Occasional reports of isotretinoin-associated HTG-induced pancreatitis have been published, presumably in subjects with underlying genetic predisposition for HTG. In several cases patients were taking other medications that may cause HTG, and these reports are complicated by the fact that isotretinoin may also cause idiosyncratic pancreatitis (112). It is therefore prudent to check for baseline HTG before prescribing these agents.

#### Propofol

Propofol is a hypnotic agent used to sedate ventilated patients in the intensive care unit. It is administered intravenously as a lipid emulsion and causes HTG in approximately 20% of patients (113). In one study, 18% of patients had triglyceride levels >400 mg/dl and in 20% of these the triglyceride levels were >1,000 mg/dl and half of this group developed acute pancreatitis (114). Since propofol infusions may deliver 24 to 48 g of fat/2 h this may be playing a significant role in the development of HTG, particularly in patients with acquired or underlying defects in triglyceride clearance (115). Propofol has also been shown to impair fatty acid oxidation (115).

#### Drugs Causing the Chylomicronemia Syndrome in Children

In a retrospective analysis of records from a large tertiary care children’s hospital ~0.1% had triglyceride levels >2,000 mg/dl and 36% of this group developed acute pancreatitis (116). Of these 14% had FCS, 19% had type 2 diabetes, 11% type 1 diabetes, 28% had acute lymphoblastic leukemia, and 14% had undergone organ transplantation on immunosuppressive medications. The children with acute lymphoblastic leukemia all received treatment with L-asparaginase and high dose corticosteroids. The development of severe HTG occurs in 4% to 19% of these children (117) and is thought to be mainly due to L-asparaginase which decreases LPL activity (118).

## Complications of the Chylomicronemia Syndrome

The most important complication of the CS is acute or recurrent pancreatitis. The development of pancreatitis in the CS is thought to be due to the local liberation of pro-inflammatory lysolecithin and free fatty acids by the hydrolysis of lecithin and triglyceride from TGL by lipases in pancreatic capillaries. Ischemic damage to the pancreas may also result from capillary obstruction due to triglyceride-induced hyperviscosity (119). Although there have been no systematic, prospective studies of the risk of acute pancreatitis in patients with the CS, the US County Health System survey of 103 patients found to have triglyceride levels >2,000 mg/dl on at least one occasion (0.14% of the health care system database), reported that hospitalization for acute pancreatitis occurred in 38% (25). In a Japanese survey of 215 patients with triglyceride values >1,000 mg/dl from a large healthcare system (0.31% of the database), 6.5% gave a history of acute pancreatitis (32). Patients become prone to developing pancreatitis when triglyceride levels exceed 1,500 to 2,000 mg/dl (120). In a retrospective analysis of a large cohort with a triglyceride measurement of >500 mg/dl, the group with follow-up triglyceride values of <400 mg/dl were associated with a significantly lower rate of pancreatitis, indicating that the lower the triglyceride levels can be reduced the less likely will be the risk of pancreatitis (121). Furthermore in a meta-analysis of 15 studies that compared patients with HTG-induced pancreatitis and those with non-HTG-related pancreatitis, HTG-related pancreatitis was associated with an increased odds ratio for renal failure (OR 3.18), respiratory failure (OR 2.88), shock (OR 3.78) and mortality (OR 1.90), thus providing strong evidence for worse outcome when pancreatitis results from severe HTG (122).

Accumulating chylomicrons are thought to be too large to penetrate the arterial well to deposit atherogenic cholesterol molecules. However, remnant particles may accumulate in patients with HTG, and these have been shown to penetrate the vascular wall and may increase cardiovascular risk in these patients (123). Due to the severity of LPL activity impairment, remnant accumulation is not a feature of FCS. Excessive circulating TRL may also deposit in parenchymal tissues such as the liver, and hepatic steatosis is associated with the development of insulin resistance and type 2 diabetes contributing to the increased prevalence of type 2 diabetes and non-alcoholic steatohepatitis (NASH) in the CS (8, 32).

### Approach to Management

The keys to management of the CS are primarily to prevent pancreatitis and secondarily to reduce the risk of CVD (**Table 2**). Although not traditionally viewed as being a target of treatment in patients with CS, prevention of NASH, the frequency of which is increased in patients with HTG, particularly if they have diabetes may also be important.

**Table 2:** Approaches to management of the chylomicronemia syndrome.

Identification and removal/management of aggravating factors	Control of diabetes
	Cessation of alcohol
	Removal/reduction or replacement of medication
Dietary management	Fat restriction; use of MCT in FCS
	Weight reduction if appropriate
Pharmacotherapy	Fibrates
	High dose omega 3 fatty acids (icosapent ethyl)
	Statins
	High dose niacin
Novel pharmacotherapeutic approaches	LPL gene replacement
	Apo C-III inhibition
	ANGPTL3 inhibition

#### Identification and Removal or Management of Aggravating Factors

If diabetes is present it is essential that optimal glycemic control should be achieved without delay. Many overweight or obese patients with severe HTG have marked insulin resistance and may require high doses of insulin to adequately control the hyperglycemia. In patients imbibing excessive alcohol it is essential that an effective alcohol cessation program be initiated. Medications that aggravate HTG should be discontinued if possible and replaced by alternatives that have less triglyceride raising properties, as discussed above. If not, they should be used at as low a dose as possible. FPLD should be ruled out by careful physical examination for lipodystrophic features. Patients without aggravating factors should be suspected of having FCS or unusual causes of CS such as autoimmune hyperlipidemia, particularly if they are relatively young.

#### Dietary Management

For the rare patient with FCS, it is essential that both dietary animal and vegetable fat intake be permanently restricted to <5% to 10% of calories (approximately 10–20 g of fat) and medium chain triglyceride (MCT) may be used to further limit chylomicron formation. In MFCS, a similar degree of fat restriction together with management of aggravating factors should be used until triglyceride levels fall to <1,000 mg/dl, at which time the diet can be somewhat liberalized, since long-term adherence to an extremely low fat diet is very difficult. Patients who are overweight will benefit from weight reduction measures and most patients, especially those with diabetes, should benefit from a high fiber diet that is low in refined carbohydrate. Although the potential benefits of bariatric surgery for severe HTG have not been formally evaluated, bariatric surgery has been shown to lower triglyceride levels, and could be considered in obese individuals (124).

#### Pharmacotherapy

Pharmacologic triglyceride lowering in MFCS and FPLD begins with fibrate therapy. This should be initiated together with management of aggravating factors and dietary fat restriction. In the United States fenofibrate is preferred to gemfibrozil because of its once daily dosage and lower rate of rhabdomyolysis when combined with statin therapy. The dose should be reduced in renal failure. The goal is to lower and maintain the triglyceride level to <500 mg/dl and this usually requires long-term therapy with the fibrate. If this is not achieved with the fibrate alone, high dose (2 grams twice daily) omega 3 fatty acid preparations can be added. Triglyceride lowering doses of niacin (2 grams daily) is a third option although this may worsen diabetes control. Individuals with FCS typically do not respond to conventional pharmacotherapy. The use of the pancreatic lipase inhibitor orlistat to reduce fat absorption was recently shown to be effective in two patients with FCS (125) and the long-term use of the microsomal triglyceride lipase inhibitor lomitapide, was effective in preventing pancreatitis in a patient with FCS although it did result in cirrhosis (126). Although there are no controlled studies, plasmapheresis may have a role to play in patients with extreme HTG and recurrent pancreatitis (127) or in pregnant women with FCS (128).

The mainstay of CVD prevention in HTG patients is statin therapy. In addition, statins may have additive modest triglyceride-lowering effects in patients with mild to moderate HTG mainly by enhancing remnant clearance. While fibrate monotherapy may reduce CVD risk, fibrates have not been shown to add benefit to statins overall, although those with moderate HTG and low HDL-C may possibly benefit (129). A recent clinical trial with the synthetic omega 3 fatty acid icosapent ethyl was shown to further reduce CVD events in hypertriglyceridemic statin-treated patients with CVD and/or diabetes (130).

#### New Pharmacotherapeutic Approaches

There has been considerable interest in developing new triglyceride lowering agents using approaches targeting LPL and its regulators because of the central role they play in TRL metabolism. None have yet reached routine clinical usage, but several appear to have considerable potential in future management of the CS and prevention of its complications.

##### Lipoprotein Lipase Gene Replacement Therapy

Alipogene tiparvovec, an adenoma-associated virus encoding a natural variant of the human *LPL* gene with higher than normal activity was developed and tested in a small number of patients with FCS (131). Although it lowered triglyceride values by 40% and there was a suggestion that the frequency and severity of pancreatitis was reduced, the effect was transient and further development was discontinued in 2017.

##### Targeting LPL Regulators

###### APOC3 Inhibition

Volanesorsen is an antisense oligonucleotide that binds to *APOC3* mRNA preventing APOC3 translation. It has been shown to lower circulating APOC3 levels 70% to 90% and triglyceride levels by 56% to 86% in clinical trials involving both FCS and non-FCS patients with moderate to severe HTG, and a reduction in the number of episodes of pancreatitis was reported (132). Although it has been approved for use in Europe, it causes thrombocytopenia. Vupanorsen is a second generation N-acetyl galactosamine-conjugated antisense oligonucleotide derivative targeting hepatic *ANGPTL3* mRNA that has greater potency allowing 20- to 30-fold lower dosing, fewer side effects and similar effects on APOC3 and triglyceride to that reported with volanesorsen (133).

###### ANGPTL3 and ANGPTL4 Inhibition

Inactivation of ANGPTL4 using a monoclonal antibody was first shown to lower triglyceride levels by about 50% in primates, but side effects blocked further development and attention has turned to inhibition of ANGPTL3. The monoclonal antibody evinacumab that blocks the action of ANGPTL3 has been found to lower triglyceride levels up to 80% in patients with mild to moderate HTG and also lowers LDL-C (132, 134). Of further importance is the possibility that both apo C-III as well as ANGPTL3 lowering may lead to reduction in CVD risk (135).

###### Other Approaches

A newly developed dual APOC3 inhibitor and APOC2 mimetic has been reported to lower triglyceride levels by 86% in experimental animals (136) and the development of an LPL-GPIHBP1 fusion protein which is highly active and resistant to inhibitors of LPL has been shown to lower triglycerides in mice (137).

## Conclusions

The clinical impact of the CS is becoming more clearly understood. Those with moderate to severe HTG are the subgroup at risk. A very large proportion of these individuals have a polygenic basis for their HTG, which is susceptible to exacerbation and to the development of the CS by common conditions such as uncontrolled diabetes, overweight and obesity and alcohol excess, as well as several widely used medications. A small proportion of CS subjects have FCS, a monogenic disorder with persistent chylomicronemia. The presence of the CS increases the risk of HTG-induced pancreatitis in proportion to the duration and severity of the HTG, and is the third most frequent cause of pancreatitis. In addition, the risks of CVD and NASH are also increased. Treatment consists of a low fat diet combined with weight reduction in overweight individuals, and pharmacotherapy with triglyceride-lowering agents, principally fibrates and high-dose omega 3 fatty acids, together with effective management of aggravating factors. Awareness of the importance and frequency in the general population of the conditions and medications that aggravate HTG should alert the clinician to the need to identify the presence of pre-existing HTG in order to effectively prevent development of the CS. Novel therapeutic agents offer hope for more effective treatment of HTG and prevention of its complications.

## Author Contributions

RG and AC reviewed the literature and co-wrote the manucscript. All authors contributed to the article and approved the submitted version.

## Conflict of Interest

The authors declare that the research was conducted in the absence of any commercial or financial relationships that could be construed as a potential conflict of interest.
